# Atypical comorbidities in a child considered to have type 1 diabetes led to the diagnosis of *SLC29A3* spectrum disorder

**DOI:** 10.1007/s42000-022-00352-3

**Published:** 2022-03-14

**Authors:** Özge Besci, Kashyap Amratlal Patel, Gizem Yıldız, Özlem Tüfekçi, Kübra Yüksek Acinikli, İbrahim Mert Erbaş, Ayhan Abacı, Ece Böber, Meral Torun Bayram, Şebnem Yılmaz, Korcan Demir

**Affiliations:** a Department of Pediatric Endocrinology, Faculty of Medicine, Dokuz Eylül University, İzmir, Turkey; b Institute of Biomedical and Clinical Science, University of Exeter, UK; c Department of Pediatric Nephrology, Faculty of Medicine, Dokuz Eylül University, İzmir, Turkey; d Department of Pediatric Hematology and Oncology, Faculty of Medicine, Dokuz Eylül University, İzmir, Turkey

**Keywords:** Monogenic diabetes, Anemia, Steroid, Tocilizumab, Intravenous immunoglobulin

## Abstract

**Introduction:**

*SLC29A3* spectrum disorder is an autosomal, recessively inherited, autoinflammatory, multisystem disorder characterized by distinctive cutaneous features, including hyperpigmentation or hypertrichosis, hepatosplenomegaly, hearing loss, cardiac anomalies, hypogonadism, short stature, and insulin-dependent diabetes.

**Case presentation:**

Herein, we report a 6-year-old boy who presented with features resembling type 1 diabetes mellitus, but his clinical course was complicated by IgA nephropathy, pure red cell aplasia, and recurrent febrile episodes. The patient was tested for the presence of pathogenic variants in 53 genes related to monogenic diabetes and found to be compound heterozygous for two *SLC29A3* pathogenic variants (p. Arg386Gln and p. Leu298fs).

**Conclusion:**

This case demonstrated that *SLC29A3* spectrum disorder should be included in the differential diagnosis of diabetes with atypical comorbidities, even when the distinctive dermatological hallmarks of *SLC29A3* spectrum disorder are entirely absent.

## Introduction

The *SLC29A3* gene located on chromosome 10q22 encodes human equilibrative transporter 3 (hENT3), a nucleoside transporter that plays a critical role in salvage pathways (1). Pathogenic variants in *SLC29A3* cause an autosomal, recessively inherited, autoinflammatory, multisystem disorder (Online Mendelian Inheritance in Man #602782), A number of conditions, including, inter alia, H syndrome, histiocytosis-lymphadenopathy plus syndrome, and pigmented hypertrichosis with insulin-dependent diabetes mellitus, previously believed to be distinct disorders, have recently been grouped within the same disease spectrum known as *SLC29A3* spectrum disorder (1, 2).

Defects in the *SLC29A3* gene cause hyperpigmentation or hypertrichosis. These distinctive cutaneous features are commonly known to be the hallmarks of the disease. Various other systemic manifestations, including hepatosplenomegaly, hearing loss, cardiac anomalies, hypogonadism, short stature, and insulin-dependent diabetes mellitus accompany the cutaneous findings (1–8).

In order to expand our understanding of this genodermatosis (9), we herein report a case, initially presenting with a clinical picture indicating diagnosis of type 1 diabetes, who developed IgA nephropathy, pure red cell aplasia, and recurrent fever episodes during follow-up.

## Case presentation

A 6-year-old Turkish boy had presented to another clinic with a 3 to 4-week history of polyuria, polydipsia, and enuresis nocturna. He was diagnosed with type 1 diabetes mellitus (T1DM) upon detection of hyperglycemia (231 mg/dl), ketonuria (ketone +1), elevated HbA1C (12.5%; normal, 4.5-6), positive insulin antibody (12.5%; normal, <8.2), and negative anti-islet cell and anti-glutamic acid decarboxylase antibodies. He was born with a birth weight of 3750 gr at full term to healthy non-consanguineous parents. His past medical history was unremarkable.

**Figure 1:**
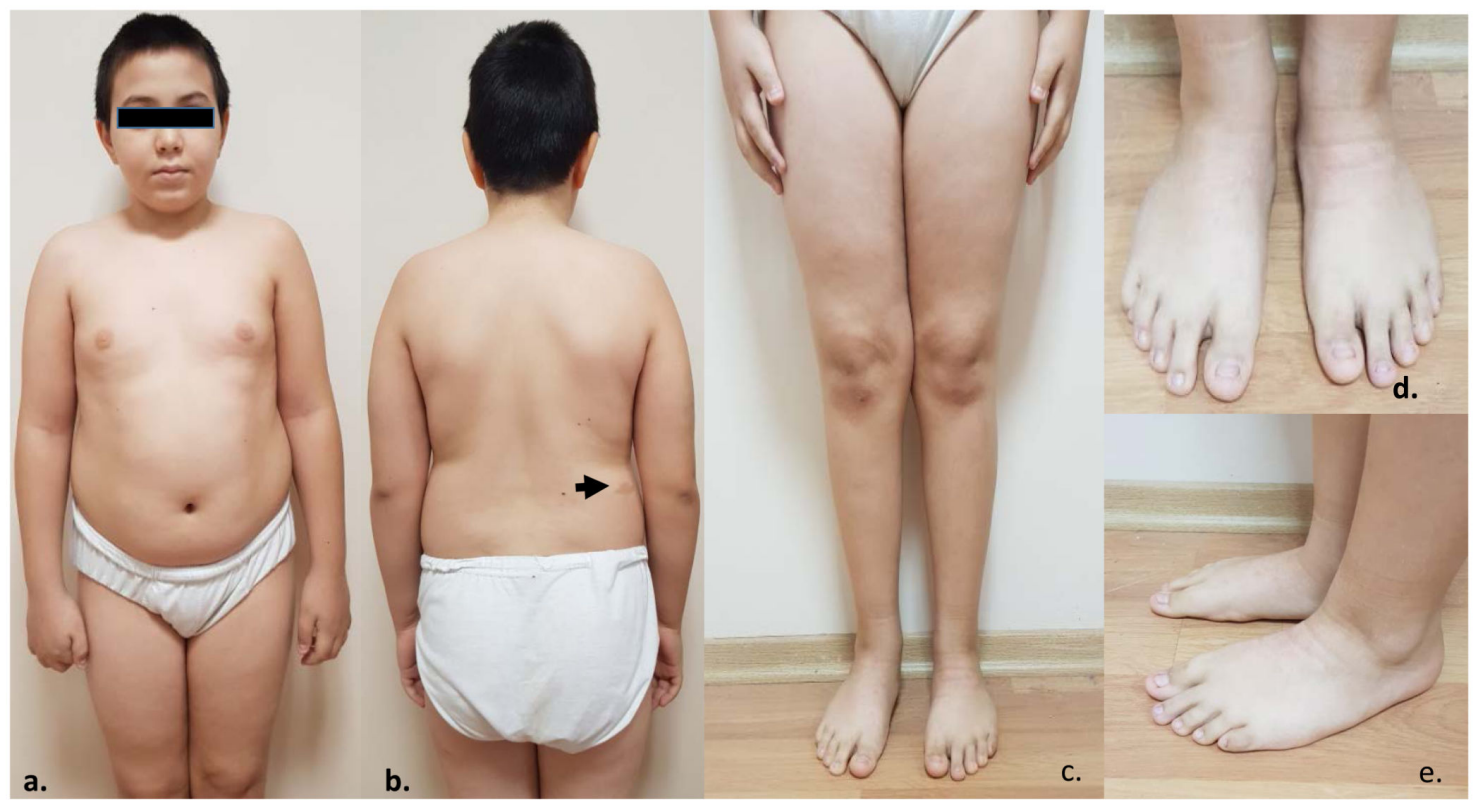
Clinical image of the patient at the age of 12.5 years. a, b. Frontal and dorsal view, arrowhead: cafe-au-lait spot. c, d, e. Clinodactyly and flat feet deformity.

On admission to our clinic after 4 months, he was on basal-bolus insulin regimen (total daily dose 0.8-1 units/kg). He had a body weight of 21 kg (0.21 SDS) and a height of 124 cm (0.1 SDS). He was prepubertal and systemic physical examination showed flat foot, fifth toe clinodactyly, and a 2x4 cm sized café-au-lait spot on the right side of his lower back (shown in Fig. 1). Liver-kidney-thyroid function tests and complete blood count were normal and celiac antibodies were negative. HbA1C level was 7.9%.

At 7.5 y of age, rashes resembling Henoch-Schönlein purpura appeared on both his legs. Laboratory studies showed no abnormality in his serum, urine, and stool. All complaints resolved within a month. There were no signs related to gastrointestinal, renal, or joint involvement. Four months later, he presented with macroscopic hematuria and hypertension (120/90 mmHg, 95^th^ centile of systolic and diastolic blood pressure in his age and height: 111/72), and laboratory analysis showed nephritic range proteinuria (37 mg/m^2^/day). Upon exacerbation of his symptoms, a renal biopsy was performed, and he was diagnosed with IgA nephritis. Prednisolone (2 mg/kg/day) and enalapril (0.2 mg/kg/day) were effective in normalizing blood pressure and renal function tests.

At age 8, he presented with pallor. His laboratory evaluation showed hemoglobin of 5.8 g/dL (normal, 11.5-17.5), total leukocyte count of 6.9x10^3^/µL (normal, 4-10x10^3^), platelet count of 379x10^3^µL (normal, 156-373x10^3^), red blood cell count of 2.7x10 ^6^/µL (normal, 4-5.7 x10 ^6^/µL) with 0.23% reticulocyte (normal, 0.5-3.5), mean corpuscular volume of 66 fL (normal, 80-95), mean cell hemoglobin concentration of 33 g/dL (normal, 32-35), red cell distribution width of 18% (normal, 12-14), and haptoglobulin of 117 mg/dL (normal 32-197). Peripheral blood smear showed non-hemolytic microcytic hypochromic anemia. Serum ferritin and transferrin saturation were 100 ng/mL (normal 23-336) and 66% (normal 7-46), respectively. Toxoplasmosis, rubella, and cytomegalovirus antibodies were negative, along with parvovirus B19 DNA PCR. Pure red cell aplasia (PRCA) was diagnosed after bone marrow aspiration. Systemic corticosteroids (prednisolone 2 mg/kg/d, tapered over 6 months), intravenous immunoglobulin (1 g/kg/d, 2 doses), and cyclosporine A (6 mg/kg/d, 2 months) did not provide any clinical improvement. Regular blood transfusion requirement necessitated chelation treatment because of elevated serum ferritin levels (1789 ng/mL; normal, 23-336).

At 11 years of age, he had four episodes of fever within 2 months, with findings of a noninfectious etiology. Acute-phase reactants were elevated, including an erythrocyte sedimentation rate of 120 mm/h (normal, <20) and CRP of 370 mg/L (normal <5). Bacterial cultures and viral serology tests were negative. Antinuclear antibody (ANA) was positive at 1:320 dilution with a speckled pattern; anti-dsDNA and extractable nuclear antigen antibody (ENA) panel were negative; genetic analysis for familial Mediterranean fever showed the E148Q variant on a single allele; adenosine deaminase-2 (ADA2) was not deficient. Abdomen ultrasonography and thoracic tomography imaging was normal. After 2 months, the fever resolved spontaneously, erythrocyte sedimentation rate returned to normal, and CRP and ANA were found to be negative.

Development of the abovementioned comorbidities that are unexpected in T1DM, weak positivity of a single diabetes autoantibody, and a low T1DM genetic risk score generated from 30 T1DM-associated common genetic variants (10.4^th^ centile of T1D reference population) prompted an investigation into monogenic causes of diabetes (10–12). Based on previous work (13), a custom-designed NGS panel including 53 genes and the NC_012920.1:m.3243A>G mitochondrial DNA variant revealed the presence of two heterozygous variants in the *SLC29A3* gene, namely, the novel variant c.890dupC, p.Leu298fs, and the previously reported variant c.1157G>A, p.Arg386Gln, the latter classified as pathogenic according to ACMG/AMP criteria (14). Sanger sequencing of the *SLC29A3* gene in the parents confirmed compound heterozygosity of our patient [Leu298fs] [Arg386Gln], since the father was found to be heterozygous for the p.Leu298fs variant and the mother heterozygous for p.Arg386Gln (Supplemental Figure 1).

On clinical examination at the age of 11 years and 10 months, he was found to have hepatomegaly with elevated liver transaminases (aspartate transaminase 178 U/L; normal, 0-50, alanine transaminase 291 U/L; normal, 0-50), presumably as a consequence of his chronic transfusion-dependence (peak ferritin at 4560 ng/mL), despite good adherence to iron chelation therapy with deferasirox (30 mg/kg/d). Other laboratory investigations were normal, including albumin 3.8 g/dL (normal, 3.8-5.4), total protein 6.2 g/dL (normal, 5.7-8), INR 1.8 (normal, 0.8-1.2), total bilirubin 0.34 mg/dL (normal, 0.3-1.2), creatine kinase 55 U/L (normal, 0-171), and ceruloplasmin 0.4 g/L (normal, 0.2-0.6); viral hepatitis serology was negative, antimitochondrial, smooth muscle and liver-kidney antibodies were negative, and the ultrasound of the abdomen showed an enlarged liver with no focal lesions. Surveillance of the derangements of liver transaminases necessitated liver biopsy to rule out hepatic involvement of *SLC29A3* spectrum disorder; the biopsy showed chronic hepatitis with massive hemosiderosis.

A laboratory investigation into other *SLC29A3* spectrum features was performed. Thyroid function (TSH 2.9 IU/L; normal 0.38-5.5, freeT4 0.95; normal, 0.5-1.5) was normal and gonadotropin levels (FSH 1.57 IU/mL; normal, 1.5-18, LH 3.01 IU/mL; normal, 0.3-6.3, total testosterone 107 ng/dL; normal, 240-827) were in accordance with his age interval. Standard screening laboratory tests of immunity, including IgA of 133 mg/dL; normal, 67-433, IgM of 50 mg/dL; normal, 47-484; IgG of 1032 mg/dL; normal, 835-2094, total IgE of 67 IU/mL; normal 0-87, total leukocyte count of 5900/µL; normal, 4 to 10x10^3^, absolute lymphocyte count of 2100/µL (1300-3500), absolute neutrophil count 3100/µL (2100-6100), and relative sizes of lymphocyte subpopulations (T cells 82%; normal, 52-78, B cells 10.5%; normal, 8-24, natural killer cells 6.5%; normal, 6-27, T helper cells 34.5%; normal 25-48, T cytotoxic cells 41.5%, normal 9-35) did not reveal any deficiencies.

Based on the results of empirical treatment trials for *SLC29A3* spectrum disorders in the literature, tocilizumab (8 mg/kg/d), a human monoclonal interleukin-6 receptor antibody, was administered (6, 15, 16). However, the anemia did not improve, and transaminase levels increased 2-3-fold after two doses of this hepatotoxic agent. Tocilizimab treatment was discontinued (Table 1). Liver enzymes returned to normal after cessation of therapy.

**Figure 2:**
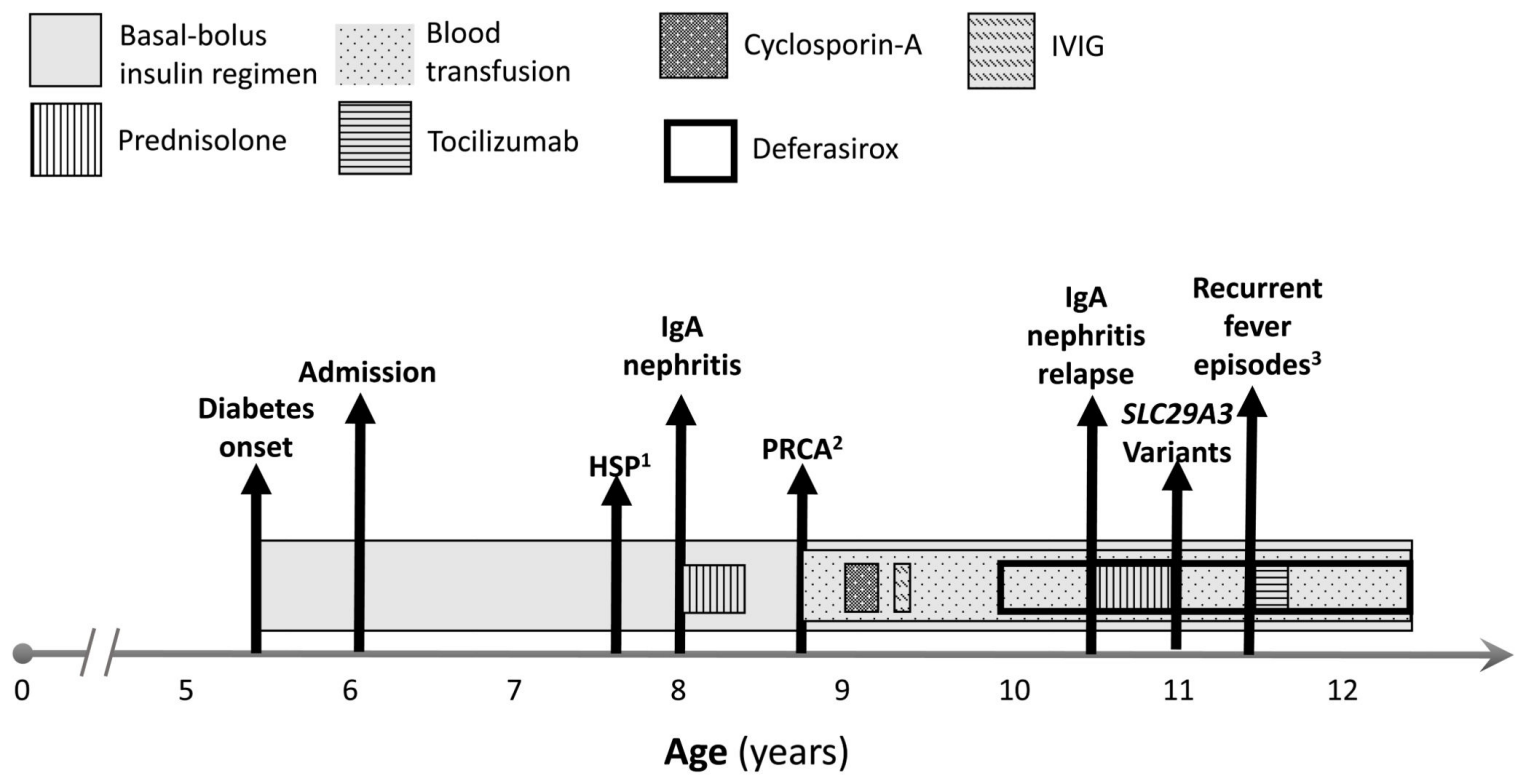
Timeline of the diagnosis and treatments. ^1^Henoch-Schoenlein purpura, ^2^Pure red cell aplasia ^3^Within 2 months, 4 episodes of admittance

Currently, at 12.5 years of age, he is on basal-bolus insulin treatment (1.5 u/kg/day) and receives erythrocyte transfusions every 3-4 weeks with deferasirox chelation (shown in Fig. 2).

## Discussion

Among several features defining *SLC29A3* spectrum disorder, cutaneous findings are considered to comprise the hallmark clinical presentation (1, 2). Almost all patients reported in the literature presented with different musculoskeletal and/or dermatological phenotypical characteristics, including hyperpigmentation, hypertrichosis, facial telangiectasias, flexion contractions, or foot deformities (2, 4, 6, 9, 15, 16). Diabetes was the first and only presenting feature in our patient. To our knowledge, there is only one other similar case without any significant dermatological or musculoskeletal involvement reported by Brostilava et al. (17), namely, the younger brother of a previously diagnosed case, a short-statured adult with telangiectasias who presented with antibody negative diabetes. In strong contrast to the previously reported cases, which were typically diagnosed after characteristic phenotypic features, including cutaneous hyperpigmentation with or without hypertrichosis and flexion contractures, our case had mild phenotypic features. He had flat feet and clinodactyly, which features were observed in 20% of all cases reported by Molho-Pessach et al. (2) and a café-au-lait spot, which was not previously reported (2, 4, 6, 9, 15, 16). Hence, despite the aggressive clinical course, characteristic dermatological involvement never emerged, which constitutes a novel finding.

In previous studies, homozygosity or compound heterozygosity for several different variants (including Gly427Ser, Gly437Arg, Arg363Gln, Arg363Trp, Arg386Gln, Thr449Arg, and Met116Arg) in the *SLC29A3* gene have been reported (1, 8, 18–21). With respect to genotype-phenotype correlation, one patient identified by Farooq et al. (20), who carried the same p.Arg386Gln variant as our patient, was reported to have hyperpigmentation, hypertrichosis, flexion contractures, heart defects, hearing loss, liver fibrosis, and elevated fasting blood glucose, while Campeau et al. (22) observed dysosteosclerosis and recurrent infections in another patient carrying the same variant. Our case, whom we found to be compound heterozygous for the two *SLC29A3* pathogenic variants (p. Arg386Gln and p. Leu298fs), had hyperglycemia, abnormalities in liver function tests, and fever episodes, similarly to the previously diagnosed cases; however, no other shared clinical characteristics were noted. There thus seems to be no genotype-phenotype correlation among the carriers of the same variant in the *SLC29A3* gene (20, 22).

The exact etiopathogenesis delineating the mechanism of diabetes remains an important subject area of research (18). The weakly positive anti-insulin antibodies observed in our case may reflect the autoimmune nature of the disorder, as suggested by several other reports (4–6, 18, 21). According to Haliloglu et al. (23), antibody positivity was 83% in T1D, but on the other side of the spectrum, McDonald et al. (24) reported detectable autoantibodies in 1-2% of all other monogenic forms of diabetes. Furthermore, Cliffe et al. (18) demonstrated that knockdowns of Drosophilia *SLC29A3* orthologs at different degrees resulted in different phenotypes with varying levels of losses, but once the insulin receptors were rescued, the intracellular signaling defect was also reversible.

Several other endocrine features were also reported in the *SLC29A3* disease spectrum. Short stature, for instance, was seen in half of the cases, and responses to treatment were best achieved after administration of supraphysiological doses of growth hormone (4, 6, 16). Despite the chronic systemic illness and recurrent doses of steroids, our patient’s height continued to be within the normal range. Another aspect, hypogonadism, observed in 16% of the cases reported by Molho-Pessach et al. (2), was also not apparent in our patient, as he showed steady progression through the pubertal stages with normal gonadotropin levels.

Different forms of immune deficiencies related to *SLC29A3* disease spectrum have also been presented in the literature (6, 16, 19, 25). Although in our case the initial immunological tests did not reveal any deficiencies and the inflammatory markers returned to normal without any need for long-term hospitalization, as was suggested by Cagdas et al. (5), further diagnostic functional analyses may be performed for cases with suspected immunodeficiencies. Congenital anomalies of the kidney and urinary tract have been reported in 6% of cases by Molho-Pessach et al. (2). However, though glomerular involvement was observed in only two cases reported by David et al. (26), a novel finding in our case was the fact that our patient had IgA nephropathy, which has not been reported to date.. Furthermore, similar to the pure red cell aplasia observed in our patient, various other bone marrow abnormalities, including pure red cell aplasia, myelofibrosis, or pancytopenia, have been reported in 10% of confirmed cases (2, 5, 27). Although there is a lack of consensus on treatment, several different treatment approaches have been tried which are reported in the literature (5, 6, 15, 25). In contrast to the adequate treatment responses reported by Bloom (6) and Cagdas et al. (5), as regards our patient, systemic steroids, immunoglobulins, cyclosporine A, and tocilizumab did not result in any therapeutic effect on bone marrow or pancreas, except for IgA nephritis, which partially showed remission under the systemic corticosteroids.

In conclusion, atypical comorbidities blurring the classical T1D course necessitate further investigation. While *SLC29A3* disease spectrum is presented as a form of genodermatosis in the literature, even when such classical dermatological hallmarks characterizing the spectrum are not evident, it should be included in the differential diagnosis of atypical courses of diabetes. The present study not only presented new features of this entity, but has also helped to expand our understanding of the spectrum of diabetes.

## Supplementary Material

Supplementary Figure 1

## Figures and Tables

**Table 1:** Response to tocilizumab treatment

	Pre-treatment	2 weeks after first dose	1 week after second dose	Normal range
**Hemoglobin (g/dL)**	13.3	10.2	7.9	13.5-17.5
**Sedimentation (mm/h)**	12	-	2	0-15
**AST (U/L)**	29	51	70	0-50
**ALT (U/L)**	65	86	104	0-50
**CRP (mg/L)**	17	-	1.3	0.2-5
**WBC (u/L)**	6300	5700	4700	4000-10000
**Urine protein/creatinine (mg/mg)**	0.3	0.26	0.21	0.15

## Data Availability

The datasets generated during and/or analyzed during the current study are available from the corresponding author on reasonable request.
