# Supplementary figures and images for: Atypical comorbidities in a child considered to have type 1 diabetes led to the diagnosis of SLC29A3 spectrum disorder
Source: Hormones (Athens). Author manuscript; Available in PMC 2022 Sep 15. (PMC7613593; doi:10.1007/s42000-022-00352-3)

1 **Supplemental Figure 1.** Images for the Sanger sequencing of **a.** the mother and **b.** the father.

2 **a.**

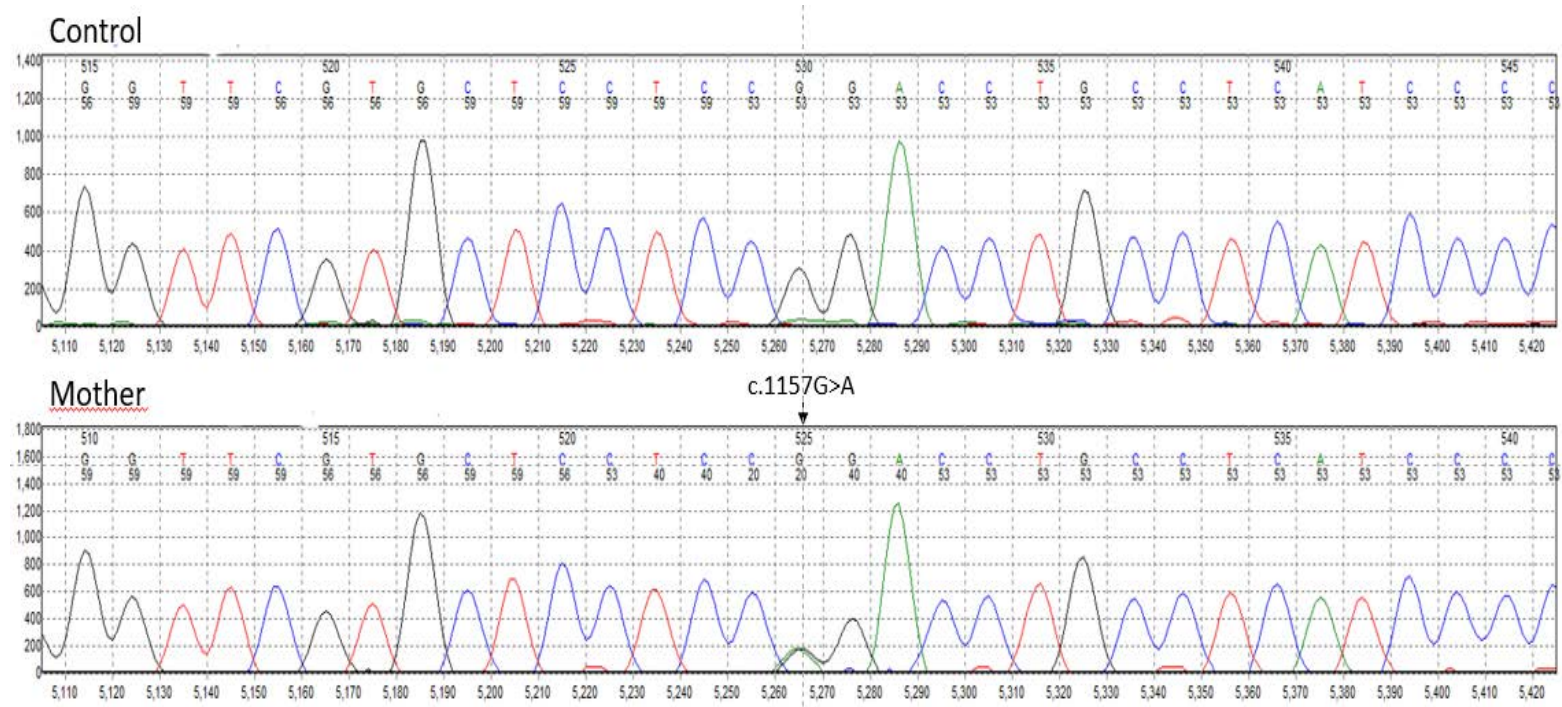

3

4

5

6

7 b.

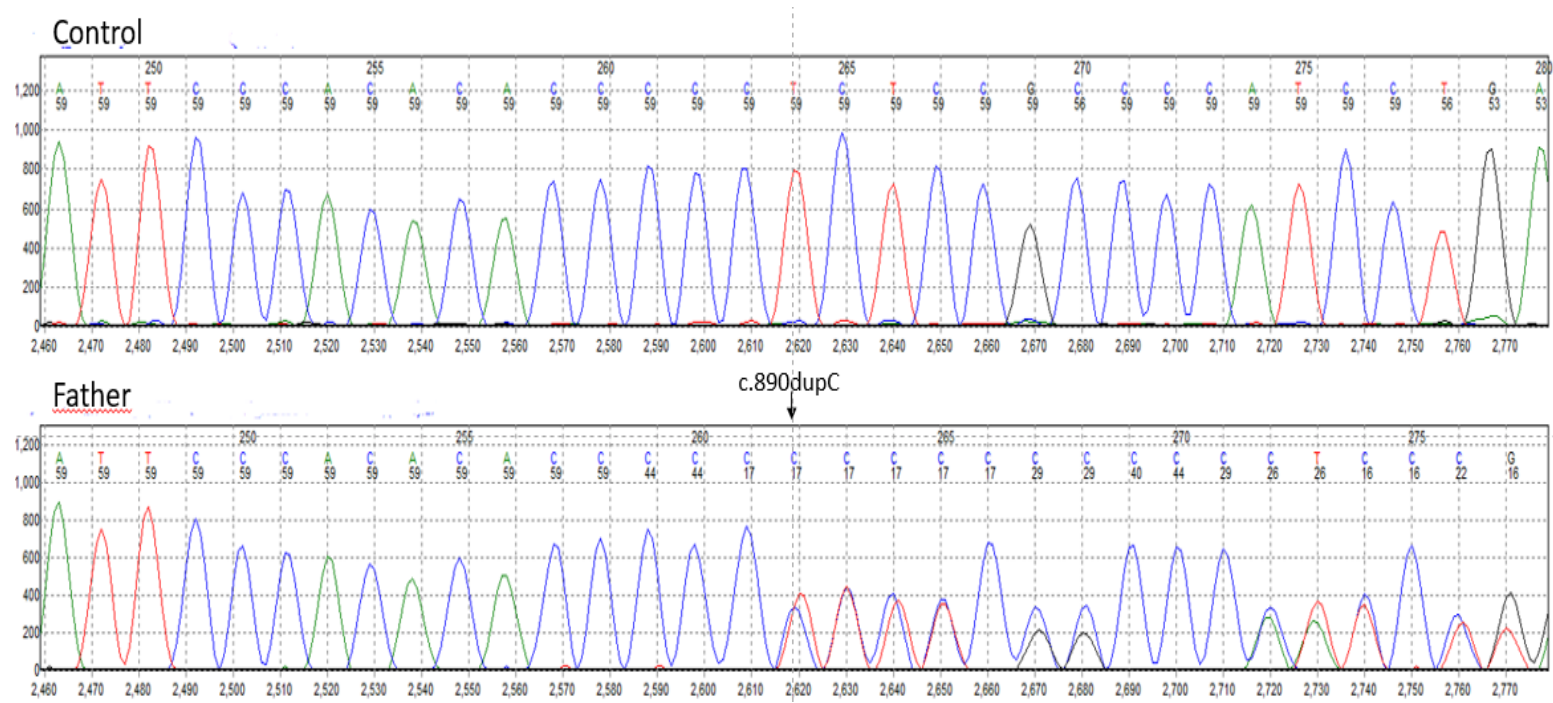

Supplement: Supplementary Figure 1 [file EMS143849-supplement-Supplementary_Figure_1.pdf]
